# Optimization-driven framework to understand health care network costs and resource allocation

**DOI:** 10.1007/s10729-021-09565-1

**Published:** 2021-05-03

**Authors:** Fernanda Bravo, Marcus Braun, Vivek Farias, Retsef Levi, Christine Lynch, John Tumolo, Richard Whyte

**Affiliations:** 1 UCLA Anderson School of Management, 110 Westwood Plaza, Gold Hall B411, Los Angeles, CA 90095 USA; 2 DataRobot, Minneapolis, MN USA; 3 MIT, Sloan School of Management, Cambridge, MA USA; 4 Beth Israel Deaconess Medical Center, Boston, MA USA

**Keywords:** Health care delivery networks, Resource allocation, Cost allocation, Optimization, Operations research

## Abstract

In the last several decades, the U.S. Health care industry has undergone a massive consolidation process that has resulted in the formation of large delivery networks. However, the integration of these networks into a unified operational system faces several challenges. Strategic problems, such as ensuring access, allocating resources and capacity efficiently, and defining case-mix in a multi-site network, require the correct modeling of network costs, network trade-offs, and operational constraints. Unfortunately, traditional practices related to cost accounting, specifically the allocation of overhead and labor cost to activities as a way to account for the consumption of resources, are not suitable for addressing these challenges; they confound resource allocation and network building capacity decisions. We develop a general methodological optimization-driven framework based on linear programming that allows us to better understand network costs and provide strategic solutions to the aforementioned problems. We work in collaboration with a network of hospitals to demonstrate our framework applicability and important insights derived from it.

## Highlights

- Industry consolidation resulted in large health networks comprising many locations of clinics and hospitals with distinct capabilities. However, by and large, networks continue to operate “together but separate”.
- Lack of operational integration results in inefficient use of capacity and potential loss of patient volume.
- A general optimization-driven framework is proposed to understand cost in health networks in a manner that informs strategic decisions around resource allocation and deployment, and network building.
- A key feature is to only consider direct variable cost and ignore ad hoc fixed cost allocations (including labor)—we capture resource usage directly through constraints instead.
- Implementation at a large health network in the U.S. to identify the best portfolio of services at each location suggests that profitability can improve by reallocating top AMC-profitable service lines to satellite facilities.

## Introduction

Over the last several decades, the health industry in the U.S. has been through an intensive trend of consolidation that has created an increasing number of large health care delivery systems and networks [14]. These networks typically consist of many locations of clinics and hospitals with distinct capabilities (e.g., academic medical centers, community hospitals, and outpatient clinics) and often have a rather complex management structure. Indeed, most health systems continue to operate “together but separate” and while they may consolidate billing, purchasing, contracting operations most of the clinical capacity issues are addressed on a facility-by-facility basis by the leadership teams of each facility. This mode of operating is the result of early consolidation efforts that were primarily driven by a desire to create market power to negotiate better *fee-for-service* prices with payers. However, the health care reform act in 2010 has fundamentally changed the underlying motivation, as well as the nature of these consolidation efforts. In particular, the ongoing shift from fee-for-service reimbursement schemes to *capitation* contracts, in which networks assume risk on managing the health of patient populations under a fixed budget and increasing number of quality metrics, creates an imperative to truly integrate care delivery networks, operationally, clinically, and financially. For instance, while under fee-for-service the network would simply miss the revenue opportunity when a patient sought care outside the network, under a capitation-like model the network may be actively penalized for the cost and corresponding outcomes associated with out-of-network care (e.g., ACO model). Fully integrated networks are better equipped to manage the care continuum by offering integrative care, and more efficiently use resources (and potentially reduce cost).

Current cost accounting practices around fixed cost allocation obscures network integration. For instance, teaching hospitals often emphasize seemingly high margin procedures, but ignore the marginal value of capacity at the network level. Wachtel et al. [30], Blake and Carter [2], Resnick et al. [25] have reported evidence of similar preferences in the allocation of resources and case-mix design in different hospital contexts and the financial measure used to inform those preferences include some allocation of labor and other fixed costs. Furthermore, poor network integration not only results in inefficient use of resources but also increases the risk of patient leakage, i.e., patients seeking care outside the network. Fibroblast [11] reports that patient leakage costs most healthcare facilities about 10% of revenue. Leakage can be driven by patients’ preferences but in many cases is due to a lack of capacity and convenience–patients are unable to schedule appointments for some procedures at specific locations or experience long wait times. Network integration requires entirely new thinking on how resources are allocated throughout the network, how to determine network objectives and optimize for them, and how to systematically understand cost and value. Unfortunately, existing practices to understanding the cost and resource allocation in health networks fall short of addressing these challenges and there is a fundamental need for more systematic approaches [13, 24].

We address the network integration problem from a strategic/tactical perspective. According to [9, 29], our problem of interest lies within the tactical level–1 year in advance when most resource capacity has been committed. The strategic decision to consolidate surgical resources across the network has already been made, and we assume resources are fixed and focus on deciding the best use of the available capacity. Operational decisions, such as staffing and scheduling, should follow the tactical allocation recommended by our model, but how to implement that is not the focus of this paper. Thus, we develop a general systematic optimization-driven framework to understand cost in a manner that informs strategic and tactical decisions around resource allocation and deployment and network building. The proposed framework is inspired by network revenue management models, specifically, linear programming optimization [27]. This approach has several major advantages over existing practices. First, unlike the existing industry practices that allocate overhead cost subjectively, the optimization-driven approach captures the notion of opportunity cost that is essential to effectively understand various important network trade-offs. An optimization-based approach does not consider each activity independently but instead optimizes them collectively given the resources that are available in the network. Second, existing practices mix the cost of building the network and the cost of ‘doing’ activities in a way that makes it quite hard to understand the incremental cost of performing an activity. In contrast, the proposed approach separates these two type of costs that we call *service cost* and *network building cost*, respectively. Moreover, it considers separately decisions related to network building and decisions related to how to best leverage the network’s capabilities. As a result, it can be tailored to inform various core strategic decisions, such as resource allocation, network building and capacity placement, and the design of location-specific and overall network portfolio of services.

To demonstrate the potential of this framework, we present the results of collaborative work with a care delivery network that is centered around a major academic medical center (AMC). We employ the model to inform, from a tactical/strategic perspective, decisions related to resource allocation and portfolio of surgical services across the facilities in the network. Existing resource allocation practices vary broadly across different hospitals and within departments of a single hospital. At our partner institution, as in many others, there is an ad hoc approach to allocate resources across the different departments (e.g., OR time) that considers profitability (e.g., net contribution margin), cost, teaching, and research needs, but it is also influenced by historical allocations and internal politics.

Other than the AMC, the network includes two community hospitals. The current state, in which each hospital manages its surgical capabilities independently, has resulted in two undesirable situations:

(i) leaked demand, which refers to patients whose care is managed by the network but somehow end up receiving surgical care outside the network, causing significant loss of revenue and/or increased costs;
(ii) imbalanced utilization of surgical capacity across the network. Specifically, while the AMC is over-utilized and does not allow growth, there is unused surgical capacity in the community hospitals.

The surgery department leadership was interested in understanding how the deployment of surgical resources and activities could allow growth in the network, and create better access in the community to recapture some of the leaked demand. As part of this effort, the leadership had to assess the portfolio of surgical services ideally provided in each network location. More specifically, the decision was centered around what procedures should be deployed to the community hospitals and what extent. These decisions have to consider the profitability of different procedures, the clinical appropriateness of performing different types of surgery throughout the network, and many other operational and clinical considerations. After extensive data collection and modeling work, which is described in detail in the subsequent sections of the paper, the resulting model was used to recommend the network how to strategically leverage the underutilized surgical capacity in the community to improve access, recapture leaked demand, and increase overall profitability as measured by *revenue net of variable cost* (RNVC). RNVC is defined as “average revenue—average direct variable cost”, where direct variable cost includes expenses that are directly attributable to the service delivered (e.g., supplies, materials, and lab tests), and that are otherwise not incurred. Note that this definition is different from the commonly used contribution margin, which includes labor cost allocations (e.g., [9, 23]).

The results of this analysis provide meaningful business insights and prescribe a concrete strategy. Moreover, many of the insights contradict common beliefs in the network that are based on both, the traditional practices of understanding cost as well as the isolated view of each hospital’s financial performance. One such insight is that some of the currently perceived profitable surgical services should be sent to the community. This stands in contrast to the belief among the leadership of the network that the services that need to be moved to the community are the ‘less profitable’. However, the current practice defines profitability based on cost allocations. This assumption does not consider the fact that a ‘profitable’ type of surgery could be blocking many other surgeries since it consumes more constrained resources, something that our model captures explicitly. Furthermore, the resulting RNVC increase from (partially) reallocating the volume of certain surgical services to the community can be quite significant; in our partner’s case, the RNVC increase is close to 12% (if all leaked demand is recovered), and we estimate that about 4% of the increase is due to the (partial) deployment of specific surgical services to the community. It is important to realize that an increase of 1% in RNVC corresponds to a much larger increase in the network’s bottom line (approx. 16%) since RNVC does not include network building costs (e.g., overhead and labor), which remain largely the same.

More generally, we believe that the proposed framework could be used to support many additional decisions around network building and resource allocation in care delivery networks. It provides an innovative way to better and more accurately understand cost in health care networks and the corresponding relationship to value. To the best of our knowledge, this is the first paper jointly modeling capacity deployment, resource allocation, and case-mix in a network of hospitals [15].

### Summary of results and contributions

Our work contributes to the understanding of cost and resource allocations, and to the practice of management in health care settings in the following ways: (1) we develop a linear programming model to capture the interaction between capacitated resources and the services that consume those resources in a multi-site network. The model optimizes the network’s objectives instead of individual departments’ goals and can be tailored to support network strategic decisions related to case-mix, deployment, and allocation of resources, and network expansion.

(2) The model provides an objective way of quantifying cost in a network environment. We distinguish between network building costs and the direct cost of performing an activity or service. This is in marked contrast to the traditional approach of (arbitrarily) allocating network building costs to activities as a way to account for their resource consumption. In our approach, capacity building costs are sunk (including contracted labor), and we model the consumption of the respective resources explicitly through constraints. This allows for an objective allocation of capacity that takes into account the opportunity cost of the resources.

(3) In collaboration with a large health network, we demonstrate how to use our framework to inform strategic decisions related to surgical activities, such as case-mix, resource allocation, and capacity placement. The network’s objective is to recapture unmet demand while maximizing its RNVC. Using real financial, inventory and capacities, and resource consumption data, we estimate the parameters of the optimization model. The estimation method could guide the implementation of our framework in different applications in health care networks. The analyses show that the network could increase its RNVC significantly by reallocating surgeries to the community (up to 12%). Finally, we contrast the model recommendations to the traditional valuation of surgical services (based on ‘net contribution’); insights from these analyses were highly valued by the managers and executives at our partner network. (4) As part of the implementation, we develop an empirical approach for mapping between the different coding systems: from DRG and ICD-9 codes to internal procedure codes. We also propose a scheme to approximately determine available resource capacity when only a subset of services is included in the model.

## Common practices to understand cost in health care

Estimating the cost of service in health care settings is challenging. The financial structure of health care networks is characterized by large fixed and indirect costs because of the typically large investments in infrastructure, workforce, and equipment. These costs are incurred to support diverse activities across the network, and cannot necessarily be attributable to services. In an attempt to better understand the cost of a service (e.g., a surgical procedure), health care networks have adopted standard principles from product cost accounting, specifically, activity-based costing (ABC). Under this method, indirect expenses (e.g., labor and overhead) are allocated to activities, and not to products, based on their resource consumption, see [5–7] for a detailed description. The use of ABC principles has brought some clarity and accountability in the understanding of costs in health care settings, [4, 12], and guidelines for the implementation of such systems in health care has been reported in [1, 28]. However, this approach to understanding the cost of providing service is not perfect. It relies on cost allocation rules that, although ideally represent the consumption of capacity, are in practice rather subjective because of the complexity in which capacity is used by hundreds (or even thousands) of different activities and services. In an attempt to understand service cost for strategic decision making, [21] studied how to allocate cost related to capital investments. Using the case of a radiology facility, they demonstrate the value of distinguishing between the cost of used and unused capacity in the cost accounting process and the impact on strategic decisions. More generally, they pinpoint the most common mistakes in the allocation of the cost of capital investments in a health care setting. Tackling the accuracy of the cost allocation rules, [17, 18] propose the Time-Driven Activity-Based Costing method (TDABC) that tailors ABC to account (using time-based allocations) for the complex resource interactions and consumption patterns in health care services. Although these improvements have made allocated costs more transparent, the following illustrative example demonstrates why they are still not appropriate for guiding tactical and strategic decisions, even in a simplified setting.

### Illustrative example

Let us consider a common capacity allocation decision at a surgery department. The operating room capacity has been increased by one additional room for the next year, and the manager has already hired a nursing team to staff the additional room. The corresponding incremental labor cost is *$*300*K* per year. The historical performance suggests that the extra operating room will effectively add 1500 operating hours per year. Thus, the labor cost per operating room hour is *$*200. For simplicity, we consider that there are only two services, I and II, and both have the same reimbursement rate, *$*1500 per case. The specific surgery duration and cost of surgery supplies, for each service, are defined in the first two columns of Table 1. Additionally, there is a floor-bed capacity for 800 days, and both services have the same length-of-stay, 1 day on average.

**Table 1:** Traditional cost allocation

Service	OR	Supplies	Labor Cost	Total
	Time [min]	Cost [$]	Allocation [$]	Cost [$]
I	120	100	400	500
II	60	200	200	400

We further assume demand arrives uniformly over time and ignore variability in arrivals and in resource consumption. In addition, patients, who cannot be seen because of scarce resources, are able to seek care somewhere else (leaked demand). The manager is interested in prioritizing the services, i.e., deciding how much operating room time to allocate to each of them in order to maximize profitability. We compare the outcome of the traditional approach that myopically allocates capacity based on services’ net profit margin (including fixed cost allocations, which includes labor) versus the optimal allocation.

Service I uses twice as much operating time as service II, hence the labor cost allocated to the former is twice as large (by TDABC). The cost allocation in Table 1 suggests that service II is more profitable (1500 − 400 > 1500 − 500), hence, a myopic manager would give higher priority to it.

Let us assume that the demand at end of the year is 500 patients type I and 400 patients type II. The manager’s priority rule would result in 400 patients for each service accepted. The floor-bed capacity would be fully utilized (50% by each service type), and the OR would be 80% utilized (53.3% by service I and 26.6% by service II). In terms of demand, 100 patients type I would seek care somewhere else (leaked demand) because of the depletion of floor-bed capacity. The revenue loss of leaked volume would be $1,500 × 100. The total profit would be *$*780*K* (400 × (1500 − 100) + 400 × (1500 − 200) − 300*K*). Unfortunately, this is not the best the manager could do. We note that at the time of deciding the priorities, the cost of the nursing team (network capacity cost) has been already committed. This cost will be incurred regardless of the type of procedures and volume performed. Thus, priority decisions should be only based on the relevant costs: those that will vary with changes in the case-mix. If instead, we define priorities to maximize the revenue net of variable cost (revenue—service cost). Then, the optimal priorities are reversed. Without changing the overall volume, the optimal capacity allocation is 500 procedures type I and 300 procedures type II, which results in a profit increase of *$*10*K*. The optimal capacity allocation is 1000/1500 OR-hours to type I service and 300/1500 to type II service, and the bed-capacity is 62.5% to type I service and 37.5% to type II service. The additional profitability in the optimal solution is not coming from a larger volume but from prioritizing the *right* kind of volume. Specifically, service type I lower service cost. Indeed, in the optimal solution, there is still leaked demand, 100 type II services, which results in the same revenue loss as before (this is because services generate equivalent revenue).

**Fig. 1:**
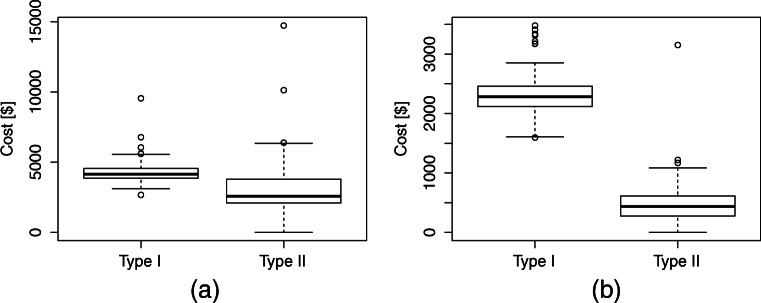
Comparison of ‘allocated cost’ vs. our service cost. Cost figures have been scaled for confidentiality. Service I corresponds to Laparoscopic Gastroenterostomy, and service II to Cholecystectomy

Following traditional cost accounting practices, the manager allocates the cost of labor to each service type based on usage (nursing hours). The problem with this approach is that the resulting ‘allocated cost’ does not capture the true cost of providing service, in particular, it does not include the *opportunity cost* of the resources consumed in the delivery of the service. Moreover, ‘allocated cost’ can make services look arbitrary more or less costly depending on the allocation rule used. In Fig. 1, we show a real example of how the inclusion of overhead and labor costs can distort the relative value of procedures. We consider two surgical services that have similar reimbursement rates and obtain the total ‘allocated cost’ (derived from the hospital cost accounting system) and the *service cost*, which does not include any labor and overhead allocations. When compared based on allocated cost (Fig. 1a), the ratio of the mean allocated cost of service I over service II is 1.29, however, when the comparison is based on service cost (Fig. 1b), the ratio increases to 4.45, making service II significantly more attractive (cheaper). This difference could influence priorities and case-mix decisions dramatically.

In reality, capacity and resource allocation decisions are significantly more complex; health care networks offer multiple procedures types in several locations. Activities and services consume several resources at different rates, incur different costs while yielding different revenues. Understanding the cost of service is critical for networks to be able to use their limited resources efficiently. In the literature, several authors have studied resource allocation in heath care settings, in particular, using linear programming. See for example [2, 3, 8, 22], and the general reviews by [16, 26], they all propose mathematical programming models to improve ‘efficiency’ in the use of resources. Although these models have proven better than ad hoc resource allocations, they typically consider a single hospital or department and include few resources in the analysis [15]. More importantly, previous models assume that the cost of service is known (typically from cost accounting), and overlook the interpretation and implications of using these cost figures for strategic and tactical purposes. Our work proposes a different approach to understand cost and inform the deployment and allocation of resources and other strategic decisions in a network environment. Specifically, we separate the cost of network building (e.g., overhead and labor), which is for the most part fixed, and the service cost, which is incurred with each additional case served. Thus, instead of allocating overhead and labor costs to activities as a way to account for resource consumption, we are going to directly model the corresponding capacity, and its consumption, through constraints within an optimization framework. In this way, our approach will capture the value of providing service by explicitly accounting for complex trade-offs and the opportunity cost of capacity in a network environment.

## Practical motivation and model

We present the business problem faced by our partner hospital network, describe our modeling approach, present the mathematical formulation, and discuss general applications of the model. For confidentiality, we omit names of the hospitals, set of services included in the study, and also re-scale strategic data, such as volume, costs, and reimbursement rates.

### The business problem: how to deploy surgical capacity in a network of hospitals?

Our partner network consists of two community hospitals and one Academic Medical Center (AMC). We worked in collaboration with a leadership team from the AMC surgical department to tackle two fundamental challenges faced by this network; (i) surgical leaked demand, and (ii) the imbalance use of surgical capacity across the hospitals in the network.

With few exceptions, it is a common practice for hospitals that are part of large networks to remain operationally independent from each other; each hospital in the network manages its own patient volumes and capacities. This is also the case for our partner network. The AMC offers the most advanced care and provides various surgical services that are not typically provided in the community setting due to limited resources, surgical expertise, and capabilities. The AMC’s surgical volume is highly dependent on referrals derived from affiliated primary care physicians within the network’s geographical area. Unfortunately, in the last few years a growing number of cases are being referred outside the network, (i). In 2012, leaked demand reached approximately 10-20% of the AMC’s surgical volume across three major service lines. Intense competition in the region and lack of timely access to care at the AMC has been conjectured as drivers of leaked demand. Thus, convenience factors such as easiness in scheduling appointments and offering services near where patients live, are becoming key differential attributes for networks to preserve their patient base. In addition, surgeons at the AMC perpetually request additional operating room time, however, hospital managers claim that operating room utilization is already at maximum levels and no additional operating room time is available. At the same time, surgical resources in the community hospitals are not fully utilized (ii). This raised the issue of whether shifting the volume of certain services, from the AMC to the community hospitals, would be an effective approach to free up capacity at the AMC. We refer to this shift in volume as *reallocation*. By doing this, the network can potentially create better access, recover leaked demand, and maximize network profitability. We refer to this as demand *recovery*. However, addressing access issues and the recovery of leaked demand require better coordination and visibility of resources across the hospitals in the network. For instance, deciding which surgical services to perform at each location depends on several factors. Firstly, not every surgical service can be performed in a community setting; hospitals in the network handle different levels of service complexity. Secondly, revenue and cost might differ across hospitals due to fluctuations in the reimbursement rates that depend on the kind of institution (academic vs. community). All this requires a systematic approach to capture complex trade-offs in order to efficiently manage capacity and deliver services according to network objectives.

### Modeling approach

In order to address the above strategic issues, we develop an optimization-driven approach. Specifically, we consider a multi-site health care network that consists of different hospitals and clinics, several resources, and various activities and services that can be performed across the different locations. The goal is to optimize *network’s welfare objectives* (e.g., maximize revenue or minimize cost across the entire network). In particular, we focus on maximizing network profitability from surgical services since this aligns with the goal of our partner network. It is important to emphasize that, in contrast to current cost accounting practices, our profitability definition only includes costs that are incurred with the realization of the service or activity, and we model the consumption of overhead and labor resources through capacity constraints. Previous work has used linear optimization for short-term case-mix planning (see review by [15]); our work differs in various dimensions. Firstly, we consider a network of hospitals and in addition to optimizing case-mix, we also decide capacity deployment of flexible resources (e.g., surgeons’ time) at each location. Secondly, our profitability measure omits labor cost, which is typically included in the contribution margin definition commonly used in the literature. In contrast, we model labor as a capacity-constrained resource. As a result, our model dissociates from subjective cost allocations and presents an objective and transparent way to understand the cost in a network environment.

**Fig. 2:**
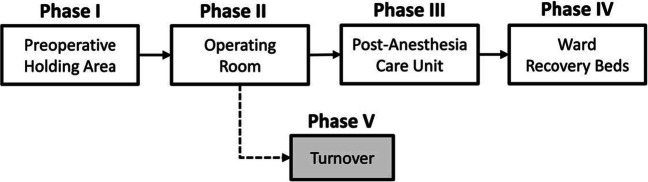
Phases of surgical path

In what follows, we describe the different components of our optimization-driven approach in the context of the delivery of surgical services at our partner network. The typical hospital path that a patient follows during a surgical care episode is shown in Fig. 2. A surgical patient physically moves along phases I-IV, but phase V is required after phase II is completed. Turnover corresponds to the room preparation/cleaning step required immediately after completion of phase II. Although the patient is not delayed by this step, accounting for the operating room downtime is important because it limits its capacity. Thus, we focus on modeling the network of resources and their capacities along with the phases of the surgical path at the three hospital locations. It is important to emphasize that in this application, and therefore in the following definitions, we focus on a subset of surgical services that are currently provided at the AMC, and not necessarily in the community. Thus, demand for surgical services corresponds to the current volume of patients served at the AMC, plus the unmet demand, which will be inferred from the leaked demand data. Furthermore, to consistently model network capacity, we restrict the AMC capacity to what is available for the set of studied procedures, plus the spare capacity that is available in the community hospitals. Later in Section 3.4, we discuss how the model can be extended to incorporate more phases, and a broader set of services and resources, as well as other applications.

**Fig. 3:**
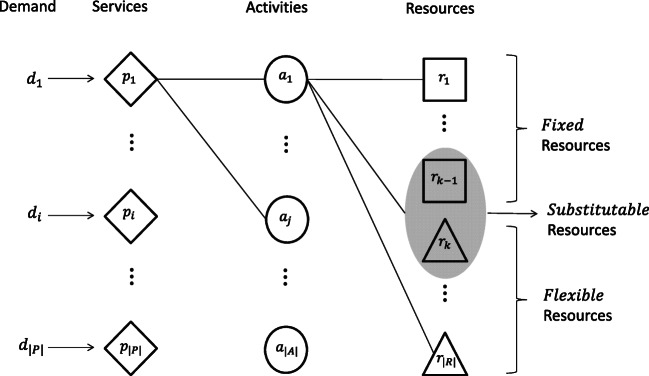
Diagram of the interaction among services, activities, and resources

- **Services.** Our partner network is interested in studying surgical services from three surgical departments (general, colorectal, and surgical oncology). To select the surgical services, we consider two criteria: (1) yearly volume is above a *minimum volume threshold*, and (2) the service is provided at least once a month. Although this selection rule is optional, it ensures that we have sufficient data to compute statistics on the usage of resources, and revenue, and cost. Infrequent services tend to have highly variable resource consumption patterns, moreover, the benefit of including them, from a managerial perspective, is unclear since they are rarely performed. Hence, we exclude them to avoid introducing noise and potentially skewing results. Using the AMC’s surgical volumes in 2012, the criteria resulted in the selection of 57 surgical services (defined based on the AMC’s internal coding system), which account for about 90% of volume across the three surgical departments.
- **Locations.** As previously described, our partner network consists of one AMC and two distinct community hospitals (COM). More generally, locations can include hospitals, clinics, and laboratories, that are part of the network where services can be provided.
- **Resources.** There are different types of resources in the network: equipment and supplies (type A), physical infrastructure (e.g., operating room, ward beds.) (type B), and staff (type C). The IT systems record exactly which equipment and supplies were used by each surgical service. Using data from AMC in 2012, we can determine a preliminary list of resources to be included in the model; Type A resources are chosen based on a *minimum usage* rule. Specifically, items that are not critical, and used in less than 5% of the cases of each surgical service are excluded. The list is reduced further by eliminating low-cost items that are included within the physical infrastructure (e.g., the surgical table is part of the operating room resource). This selection process results in a final list of 197 type A resources which were reviewed and approved by the AMC nursing team. Type B resources correspond to operating rooms, preoperative and post-anesthesia bays, and ward beds. Type C resources broadly include nurses, anesthesiologists, and surgeons. Type C resources are assumed to be staffed on an aggregate per operating room basis: a common practice in many hospitals. Preoperative and post-anesthesia bays also follow a similar staffing model for nurses. Thus, having an operating room available in our application means that the surgical team (except for the surgeon) is guaranteed for that room. Hence, the only type C resource that is explicitly included in the model is the AMC surgeons.
  In the model, we also distinguish between *fixed* and *flexible* resources. *Fixed resources* are tied to a particular location and can only be used to deliver service at that location (e.g., surgical supplies, operating room, etc.). *Flexible resources*, on the other hand, are shared across the network, and can potentially be mobilized from one location to another (e.g., AMC surgeons who spend two days in the community hospital and three days in the AMC). Furthermore, we also identify a subgroup of *substitutable resources* that can be safely exchanged for each other (e.g., two orthopedic surgeons with overlapping surgical capabilities). In this application, we assume that resource types A and B are fixed to a specific location, while AMC surgeons (resource type C) are flexible and substitutable (based on technical skills). Indeed, a distinctive feature of the model is to decide how to deploy surgeons operating time across the different hospitals in the network.
  The idea of modeling surgeons as a substitutable resource derives from the observation that surgeons have overlapping skills, moreover, each surgeon can provide several surgical services. To identify ‘who can provide what’, we create a classification of services and surgeons based on historical data. With the assistance of the surgeons from our partner network, we classify each surgical service as one of seven specialties (bariatric, breast, colorectal, endocrine, esophageal, general, and Hepato-Pancreato-Biliary (HPB)), and develop the concept of *surgeon classes*. A class is a set of specialties so that each surgeon belongs to exactly one class, and two surgeons are in the same class if they can perform surgeries from the same set of specialties. Thus, each surgeon class is treated as a different resource in the model, which facilitates tracking surgeons’ capacity and the consumption of it. For the set of studied surgical services, we have 20 surgeons, 7 specialties, and 10 surgeon classes.
- **Capacity of resources.** The capacity of resources is determined by location and measured in *time equivalent units*. That is, capacity depends on the total number of units of resource available at each location, and the typical number of hours per day that the resource is available. Thus, the daily capacity of a resource at a specific location corresponds to *the availability (hours/day)*× *the quantity of resource (units)*. In general, resources are assumed to be available during the normal operating hours of the respective hospital. For example, preoperative bays and operating rooms are usually available between 9-10 hours a day, while post-anesthesia beds are usually available for 10-12 hours a day. Ward-beds are available 24/7. Supplies, equipment, and staff generally follow the daily availability of the operating room. The daily estimate can be adjusted to obtain monthly or yearly estimates of capacity by simply multiplying by the number of days in the model horizon. A potential issue with this approach, however, is that it assumes that the entire resource capacity is available for the set of studied services. In reality, resources are also used by several other services that are not part of our model. Limiting the analysis to a subset of surgical services introduces a unique challenge in capacity modeling. To reconcile the discrepancy, we approximately segment the capacity into the portion that is available for the studied procedures by looking at historical utilization. The details of this approach are described in Appendix A.
- **Activities.** To model the consumption of resources, we introduce the concept of *activities*. These are specific actions required for the completion of the service. In general, services, resources, and activities are connected in the following manner: each service requires the execution of various activities in order to be completed, and each activity consumes a certain bundle of resources in order to be executed. Figure 3 illustrates the interaction between services, activities, and resources.
  We consider activities as the phases of the surgical path. For example, to provide a breast cancer surgery (service), the activity of delivering pre-operative care (Phase I) requires a bed, equipment, nurse time, etc. for the duration of the phase. The next activity is the surgery itself (Phase II) and requires an operating room, a surgeon who can provide the breast surgery, and equipment for the duration of the phase. Note that some activities are demanded by various services (e.g., pre-operative care activity), however, the resource consumption may vary depending on the service (e.g., the typical operating room time needed for a transplant is much longer than for an appendectomy surgery).
- **Resource usage.** The amount of resources used by a specific service is determined by the activities required for the completion of that service and their corresponding resource consumption. Similarly to the modeling of capacity, we define resource usage in time equivalent units. Different patients may require a different amount of resources; we consider typical resource usage. This corresponds to the *typical quantity of resource used*× the *typical duration of the activity that uses that resource*, and we assume that resources are used during the entire duration of the activity. Specifically, the *typical activity duration* is modeled as the median of the duration of the phase I–IV. The hospital standard turnover time is used for phase V. The *typical resource quantity* is modeled as the average amount of resource used in each phase. Details available in Appendix A.
- **Demand for services.** Modeling demand requires us to quantify current volumes, as well as the ability to grow demand for specific surgical services. The leakage phenomenon can be used to obtain a reasonable estimation of the network’s opportunities to grow. Thus, we consider two sources of demand; *existing demand* and *unmet demand*. Existing demand corresponds to the AMC volumes for the set of studied services. This is considered as the baseline demand. Unmet demand is estimated by analyzing claims data of in-network patients that received surgical care outside the network. To the best of our knowledge, we are the first work informing unmet demand based on leakage data. One of the challenges that we faced in estimating unmet demand, is that the leakage data is indexed using a different coding system, Diagnosis-Related Groups (DRGs). The challenge is that multiple DRGs are assigned to a single surgical service and vice versa. To resolve this, we create a data-driven mapping based on the empirical frequency of DRGs on existing demand and use this to obtain an estimate of unmet demand per service type. The mapping approach is described in Appendix A.
- **Revenue and cost.** For each surgical service performed, the network collects some revenue and incurs some cost. These quantities may vary across patients due to insurance differences, intensity of care delivered, and payment delinquency. The model considers typical revenue and cost figures as the average payment collected and the average cost incurred with the realization of the service per location. One of the challenges we encountered in estimating average revenue per surgical service was that financial data is, as it is in many hospitals, recorded by patient encounter (i.e., entire episode of care), and not by surgical service. Moreover, within each encounter, single charges are listed using ICD-9 procedure codes. Thus, figuring out the revenue generated by each surgical service is not straightforward. The problem is that ICD-9 codes do not map to the internally used surgical service codes uniquely. To overcome this, we create a mapping based on the empirical occurrence of ICD-9 codes on surgical service codes, and used this to determine the average revenue per surgical service at each location. Details available in Appendix A.
  From the cost side, we distinguish between two sources of cost; the *network building capacity cost* and the *service cost*. The former includes the costs attributable to infrastructure, equipment, and labor, which determine the operational capacity of the network. The second source of cost corresponds to expenses incurred with each extra unit of demand served (e.g., supplies, medications, and disposable kits). In economic terms, the first group of costs includes fixed and indirect costs, while the second one only encompasses direct variable costs. We notice that, given a fixed level of capacity, the first group of costs is committed (sunk), and will be incurred regardless of the actual combination of services performed. Hence, we will not include them in the model. Similar to the revenue data, cost data is also indexed by the encounter and ICD-9 codes, but further broken down into specific cost departments. This level of detail allows us to distinguish surgical service cost from (discretionary) allocations of network overhead costs; we only consider the former one in the computation of average service cost. The estimates are based on the AMC cost records in 2012, and we assume that the cost in the community hospitals is the same.

These components are the building blocks of the mathematical formulation presented in the next section.

### Mathematical formulation

The model considers a fixed time horizon over which it decides on the volume of each service to be offered at each location to maximize the average revenue net of variable cost (RNVC), subject to specific business and operational constraints that ensure that a particular combination of services can be performed in practice. Notice that the RNVC metric includes the sum of average revenue—average service cost (*π*_*pl*_) for all services, which does not include any of the (discretionary) cost allocations related to overhead and labor (network building capacity costs). Instead, the capacity and consumption of these resources are modeled through constraints. It is important to note that an increase in RNVC corresponds to a much larger increase in the network’s bottom line (total revenues—total costs); a first-order estimation suggests that 1% increase in RNVC corresponds to approximately 16% increase in the network’s bottom line (this assumes network margin is 4%, and overhead and labor are 60% of total cost).

We consider two sets of decision variables; *x*_*pl*_: number of cases of service *p* to be performed at location *l*, and *y*_*ral*_: amount of substitute resource *r* allocated to activity *a* at location *l*. The second set of variables allows us to model the deployment of capacity for resources with substitutes that are also flexible, i.e., that can be allocated to various sites and to avoid double allocation. Observe that since we are addressing the network optimization from a strategic perspective, the decision variables are assumed continuous without affecting the interpretation of the optimal solution. The formulation of the network optimization problem is as follows

1 $$ \begin{array}{@{}rcl@{}} & \underset{\mathbf{x, y}\geq 0}{\max} & RNVC=\underset{l\in L}{\sum}\underset{p\in P}{\sum} \pi_{pl} x_{pl} \\ & \text{s. t.} & \underset{p \in P}{\sum} \underset{a \in A}{\sum} u_{parl} x_{pl} \leq c_{rl} \quad \forall r \in R^{Fix}, l \in L \end{array} $$

2 $$ \begin{array}{@{}rcl@{}} & & \underset{l \in L}{\sum} \underset{p \in P}{\sum}\underset{a \in A}{\sum} u_{parl} x_{pl} \leq \tilde{c}_{r} \quad \forall r \in R^{Flex} \end{array} $$

3 $$ \begin{array}{@{}rcl@{}} & & \underset{p \in P}{\sum} u_{parl} x_{pl} \leq \underset{s \in S(r)}{\sum} y_{sal}\quad \forall r \in R^{Subs}, \\ & & a \in A, l \in L \end{array} $$

4 $$ \begin{array}{@{}rcl@{}} & & \underset{a \in A}{\sum} \underset{l \in L}{\sum} y_{ral} \leq \hat{c}_{r} \quad \forall r \in R^{Subs} \end{array} $$

5 $$ \begin{array}{@{}rcl@{}} & & {{\varDelta}}_{p}^{-} \leq \underset{l \in L}{\sum}{x_{pl}} \leq {{\varDelta}}_{p}^{+} \quad \forall  p \in P  \end{array} $$

6 $$ \begin{array}{@{}rcl@{}} & & \delta^{-}_{pl} \leq x_{pl} \leq \delta^{+}_{pl}\quad \forall p \in P, l \in L \end{array} $$

The model notation is summarized in Tables 3 and 4 in Appendix A. The constraints Eqs. 1 and 2 ensure that the amount of required resources does not exceed the available capacity at each location (fixed resources) and across the network (flexible resources). The left-hand-side (lhs) adds up the total amount of resource across services, activities, and locations (only for Eq. 2) required to serve the optimal volumes. The right-hand-side (rhs) accounts for the total capacity of resources available at each location in Eq. 1, and across the network in Eq. 2. The third constraint guarantees the correct allocation of substitute resources across activities and locations. For each substitute resource, the lhs adds up the total amount of resources required to execute a specific activity across services at a specific location. The rhs corresponds to the total capacity that the model will assign, from the pool of substitute resources to the specific activity and location. Since substitute resources are modeled as flexible resources, constraint Eq. 4 guarantees that the allocated capacity across activities and locations does not surpass the network resource capacity for each substitute resource. We note that constraints Eqs. 2–4 serve to model the deployment of the capacity of surgeon’s time across locations while taking into account surgeons’ capabilities to perform a surgical service through substitution among them. Finally, constraints Eqs. 5 and 6 are demand related. We consider that the volume that the network, and each hospital, serves is within some minimum and maximum limits. Constraint Eq. 5 captures the extent up to which the network can decide on the combination of services to offer. For instance, the network cannot just focus on the most profitable services, it must offer a wide variety, even less profitable ones, to cover the needs of its population. Krishnan et al. [20] shows empirical evidence on how merging hospitals tend to redeploy resources to focus on high-profit services, but they still maintain a share of non-profitable service lines. Similarly, constraint Eq. 6 is used to ensure diversity in the portfolio of services offered at each location, and to control for the reallocation of demand among hospitals. In practice, we impose these limits to avoid recommendations that suggest converting hospitals to uniform services (this has been observed in practice, see [10]).

In Section 4, we apply this model to our partner network on a subset of surgical services that are currently performed at the AMC. The leadership team wishes to understand how to efficiently use spare surgical capacity in the community hospitals to recover some of the unmet demand. Specifically, they need to inform the deployment of surgeons’ capacity across the network and case-mix at each location. The parameters of the model are estimated to represent the current resource usage, the current AMC capacity available for the set of studied services, and the community hospitals’ spare surgical capacity. Demand is determined using estimates of existing volumes and unmet demand, which is estimated based on leakage data.

### General applications

The formulation of the network problem is quite general and can be tailored to study a wide range of network strategic decisions. For instance, optimal volumes can be used to determine capacity budgets to prospectively allocate capacity among services across the network. The modular structure of the model is so that its scope can be easily extended by, for example, incorporating more resources, activities, services, and locations. Moreover, the model objective can be adapted to other *network’s welfare objectives*, e.g., maximizing profit, throughput, access, or minimizing cost, or any combination of them. More generally, the network optimization model can support strategic decisions related to network integration, expansion of service lines, recovery of leaked demand, and improvement of access to care. Specifically, applications include:

- Business development: Evaluate how to better use spare capacity across the network; which services to offer at each location. Determine the priority in which types of leaked services should be attacked; which leaked demand is most valuable for the network.
- Operations: Determine the extent to which the network can meet (or not meet) expected demand with the existing resources, and how capacity should be deployed. Evaluate the financial impact of shifting capacity across the different network locations. Identify which resources are limiting and whether it makes financial sense to expand capacity (network building decisions).

Expanding on network building decisions, our model can be used to evaluate the marginal benefit of capacity investments by simply looking at the shadow prices of the resources. Also, it can inform operational decisions. The capacity and resource allocation in the short term is difficult due to variability in demand arrivals, duration, and resource consumption. Thus, solutions at the aggregate level need to be translated into an operational feasible plan, e.g., for scheduling and staffing. The formulation presented above can be easily adapted to account for operational variability. For instance, by using more conservative estimates of the typical resource consumption parameters. For instance, instead of using the mean or median resource consumption as the typical amount, we can simply use higher quantiles. Alternatively, we could also add or modify constraints, for example, by adding buffer capacity to account for the variability in duration, or by reserving capacity for emergency cases, and so on. With these simple adjustments, we can obtain solutions that can better complement and guide short-term operational decisions.

## Application and discussion of results

We employ the above model to recommend how to use the network surgical spare capacity to our partner network. The analyses focus on identifying which services to shift to the community, what the best use of the spare community capacity is, and how this capacity should be allocated to the AMC’s surgeons. We also analyze changes in utilization as a proxy for access improvement. The time frame studied is one year, and the parameters of the model are estimated according to this timeline. The parameter estimation procedure is described in detail in Appendix A. The recommendations of the model are compared to the recommendations resulting from the traditional ‘net contribution’ approach. As described in Section 2, this commonly used approach values services by revenue—‘allocated cost’, where allocated cost includes the (discretionary) portions of overhead and labor costs that are traditionally assigned based on cost accounting principles. The ‘net contribution’ approach closely represents how executives and managers value and prioritize services in our partner network, and generally in the industry. Specifically, we study the following scenarios:

- **Baseline** (Section 4.1). This represents the current operations at the AMC, and we use it as a reference for subsequent analyses. Specifically, we consider that all existing demand is served at the AMC, and unmet demand and spare capacity in the community are excluded in this scenario. The validation of this scenario is based on the inputs from practitioners.
- **Estimate the value of capacity at the AMC** (Section 4.2). This study quantifies the benefit of having an optimal case-mix from an RNVC perspective; can the AMC increase the value generated out of its capacity by choosing an appropriate case-mix? If so, which services should be emphasized to maximize RNVC? In the analysis, we consider the baseline scenario as a starting point and allow for small changes in volume while maintaining the same levels of resource utilization.
- **Quantify the value of recovering leaked demand using spare network capacity** (Section 4.3). This analysis answers the question on how to best use the spare capacity in the community to increase the network’s RNVC. Which services should be *reallocated* from the AMC to the community, and how unmet demand should be *recovered* across the network? Specifically, the study expands upon the baseline scenario by including spare capacity in the community hospitals and unmet demand. We assume that some portion of the baseline AMC demand can be reallocated to the community and that leaked demand can be recovered across the entire network, provided that the necessary resources exist. In addition, we restrict the utilization of AMC bottleneck resources to the baseline utilization. The spare capacity in the community is fixed, except for the ward-beds, for which we explicitly report the capacity needs based on optimal volumes and typical length-of-stay.

### Baseline

**Fig. 4:**
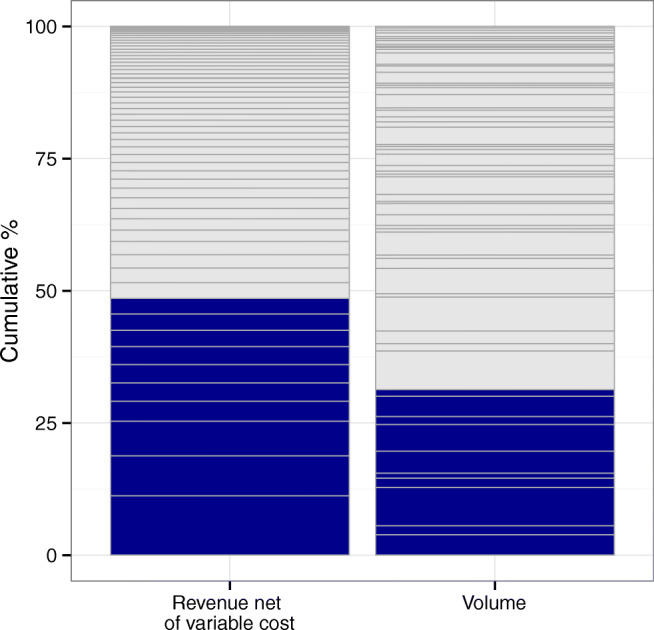
Baseline cumulative revenue net of variable cost and volume for the set of 57 studied services. Note: Stagged bars represent different service lines. The dark bars correspond to the top ten largest contributors of RNVC. Data from 2012

In the baseline scenario, we focus on the AMC’s current operations excluding the community hospitals’ capacity and network’s leaked demand. Later, in Section 4.3 we study the value of using the entire network capacity to recover leaked demand. Thus, we set the model resources capacity and demand parameters according to the AMC’s current capacities and existing demand levels. To better understand the importance of the different services at the AMC, Fig. 4 shows the cumulative RNVC and corresponding volume. We observe that 10 services account for 50% of the RNVC, while their volume accounts for about 31% of the total volume across the 57 studied services.

To validate that this scenario represents the current operations at the AMC, we obtain utilization values from the output of the optimization model. The operating room has the largest utilization, about 82%, followed by 62% and 35% in the preoperative and post-anesthesia bays, respectively. The overall surgeon time utilization is 62%. For the ward-beds capacity, we did not have a hard capacity constraint in the model, but we consider a lower bound based on the existing volumes and typical length-of-stay. The data suggests a minimum of 26.23 beds per year to serve the existing demand. Based on our conversations with the managers at the surgical department, these results are a reasonable representation of the current AMC utilization levels. Moreover, the surgical team also validated that the operating room utilization is already high, and cannot be increased without negatively affecting the service quality (e.g., excessive overtime and delays in the schedule). Based on their knowledge, the team also considers that ward-beds are a limiting resource. Conversely, they believe that surgeon time utilization could still be increased. Note that this, and the issue of leaked demand and spare surgical capacity in the community hospitals, motivated our work, to begin with. Therefore, the bottleneck resources are the ward-beds and operating rooms. In the subsequent analyses, we restrict the utilization of these AMC resources to be at most the baseline utilization. The model also suggests that most of the resources, especially equipment, have very low utilization, hence we do not adjust their utilization since they are not significantly constraining volumes. However, we do recognize that the coordination and scheduling of these resources will be crucial to ensure case-mix feasibility at the operational level.

**Fig. 5:**
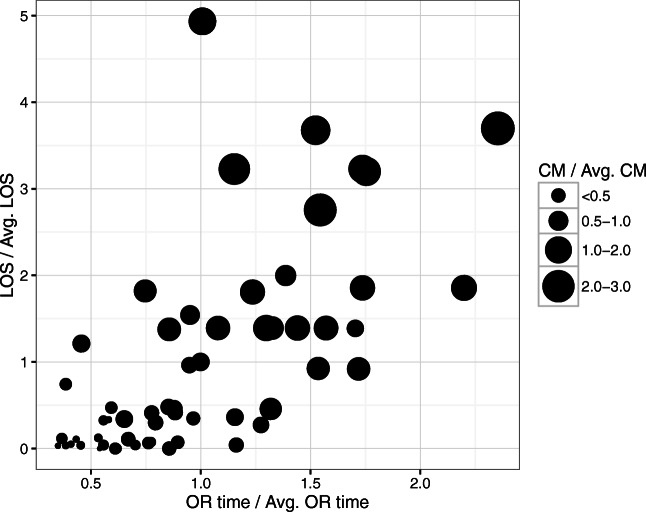
Comparison of resource consumption and value by service. Note: Each point represents a different service line. The x-axis an y-axis correspond to the ratio of operating room and ward-beds consumption over the average consumption, respectively. The bubbles size represents the ratio of profitability (revenue—service cost) relative to the average profitability. Averages are computed on the set of studied services

We note that surgical services can be very different from each other. Figure 5 shows a comparison of the different services based on their resource consumption and profitability (revenue—service cost). Generally, services that consume more resources than the average service, also tend to report higher profitability, which is represented by larger bubble sizes.

At a more aggregate level, the medical specialties with the largest contribution to RNVC are general, colorectal, and breast, there are also the specialties with the largest volumes (Table 2). In terms of average profitability per case, the specialties are ranked as HPB, esophageal, colorectal, bariatric, general, endocrine, and breast.

**Table 2:** Sub-specialty profitability in baseline scenario. Percentages computed over the 57 service lines included in the study

Sub-specialty	% Revenue net of variable cost	% Baseline volume
Bariatric	4%	4%
Breast	12%	21%
Colorectal	27%	22%
Endocrine	9%	10%
Esophageal	2%	1%
General	36%	39%
HPB	11%	3%

### Estimate the value of capacity at the AMC

**Fig. 6:**
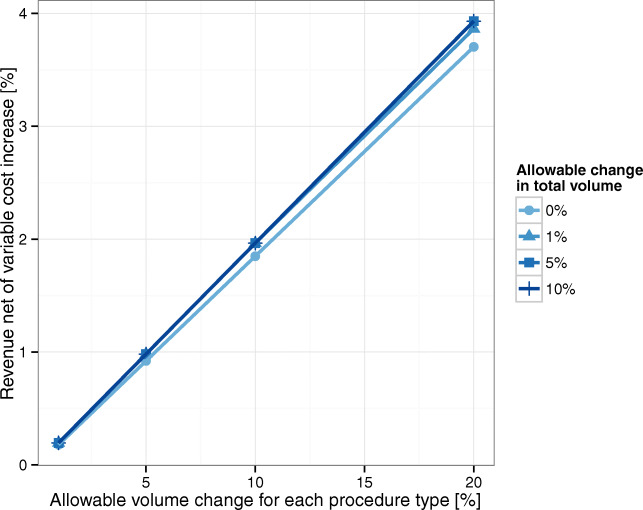
AMC’s RNVC increase for small changes in services volume. Note: The different curves are closed to each other because the AMC is capacity constrained. Thus, even if we allow to increase total volume, the AMC does not have the capacity to serve it

Starting from the baseline scenario, we allow the model to make small changes in AMC volumes and evaluate the corresponding impact on profitability. Specifically, for each service, we allow an ± *x%* volume change (i.e., we modify bounds in constraint Eq. 6 by (1 ± *x%*)), while the total volume stays within ± *y%* of the existing volume (i.e., we modify bounds in constraint Eq. 5 by (1 ± *y%*))). Figure 6 shows the potential RNVC gains for different changes in services volume. By changing services volume (x-axis) in up to ± 5*%*, a 1*%* increase in RNVC can be obtained. We recall that a 1% increase in RNVC will approximately result in a 16% increase in AMC’s bottom line.

**Fig. 7:**
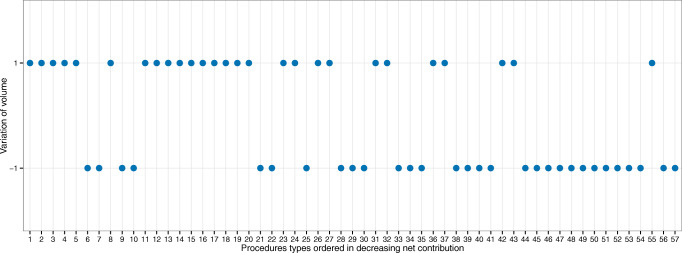
Changes in AMC’s portfolio of services. Note: Directional volume changes are represented by ‘ + 1’ when positive and by ‘− 1’ when negative. We assume that the volume of each service can change in up to ± 5*%*, and total volume stays within ± 1*%* of the total baseline volume

We also analyze the consequent changes in the portfolio of services at the AMC. Figure 7 shows directional changes in volume for each service. The services are ranked based on their ‘net contribution’, which is the traditional approach for prioritizing services based on profitability. The y-axis notation, ‘ + 1’ and ‘− 1’, indicates directional changes in AMC volume (increase (‘+ 1’) or decreased (‘− 1’)) when the optimization model is allowed to slightly vary baseline volumes by up to ± 5*%*. Intuitively, ‘ + 1’ indicates that prioritizing that service at the AMC will expand network profitability. In contrast, ‘− 1’ implies that higher profitability could be obtained by reducing that service’s volume. This marginal analysis allows us to see that the current volumes and profitability prioritization are not optimal at the AMC–higher profitability could be achieved by de-emphasizing the volume of some services in the top 10 net contribution ranking.

Decreasing the volume of services 6 (General surgery), 7 (HPB), 9, and 10 (Colorectal) were somehow counter-intuitive to executives and managers at our partner network. The discrepancy between the model and their ranking is because the model explicitly accounts for services’ resource consumption while maximizing RNVC. For example, service 6 reports high ‘net contribution’, however, it is also a very expensive service in terms of the usage of bottleneck resources (highest bubble in Fig. 5). The problem with the ‘net contribution’ ranking is that it does not capture the interaction among services that compete for limited resource capacity. Thus, by reducing the volume of service 6, the model is freeing capacity that is then used to increase the volume of several other seemingly ‘less profitable’ services (e.g., 41 (General surgery), 43 (Colorectal)).

### Quantify the value of recovering leaked demand using spare network capacity

We optimize the network under two changes: (1) allow reallocation of existing volumes from the AMC to the community, and (2) allow recovery of leaked demand across the network. Note that we do not restrict where to recover unmet demand, the model chooses the best location for it. Providers believe that a small volume reallocation from the AMC to the community is operationally feasible, as long as the resulting case-mix can be accommodated within the AMC capacity limits. Estimates of leaked demand volume for each facility is obtained from historical claims data by assigning patients to the closest hospital in the network according to patients’ home zip code. We consider different scenarios of leaked demand recovery. Ideally, the network would like to recover 100% of leaked demand, however because of capacity limitations and some patients’ strong preferences, it might not be able to do so. Alternatively, one might interpret leaked demand recovery as a proxy for low-hanging-fruit growth opportunities. Hence, recovery of unmet demand in our model is a way for the network to identify which growth opportunities to pursue and which surgical procedures to prioritize at each facility. Thus, the different scenarios aim at capturing the network’s effectiveness in retaining patients in-network. Specifically, we modify demand bounds (in constraints Eqs. 5 and 6) to allow reallocation of volume from the AMC to the community hospitals of at most *x%* for each service and recovery of unmet demand across the network of up to *w%* of leaked volume for each service.

**Fig. 8:**
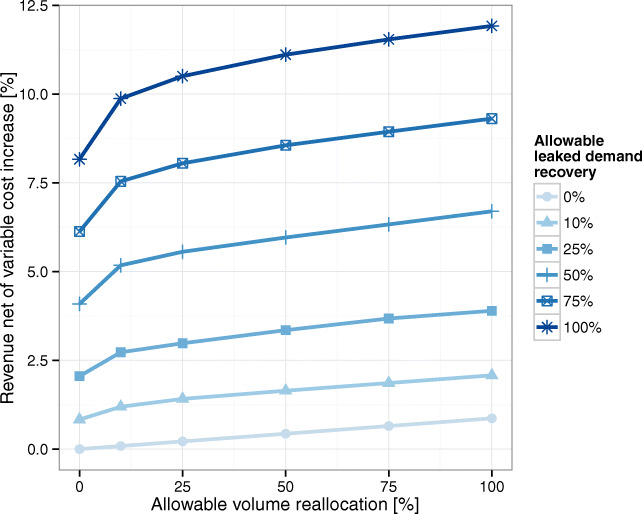
Network RNVC increase obtained by reallocating AMC volume and recovering unmet demand

In Fig. 8, we observe that by simply reallocating volumes (i.e., 0% leaked demand recovery curve), the network can increase RNVC by up to 1.2%. This gain is simply reflecting differences in reimbursement rates across locations. Once again, we recall that a 1% increase in RNVC corresponds to approximately 16% in the network’s bottom line. Now, if the network is able to backfill AMC reallocated volumes by recovering leaked demand, RNVC can be increased by up to 12% (100% AMC volume reallocation and 100% leaked demand recovery curve). Additionally, we also observe that reallocation has decreasing marginal returns; hence, small reallocation of AMC volumes can provide most of the benefits. Finally, notice that these gains do not require additional capacity at the AMC since spare capacity in the community hospitals is used instead.

**Fig. 9:**
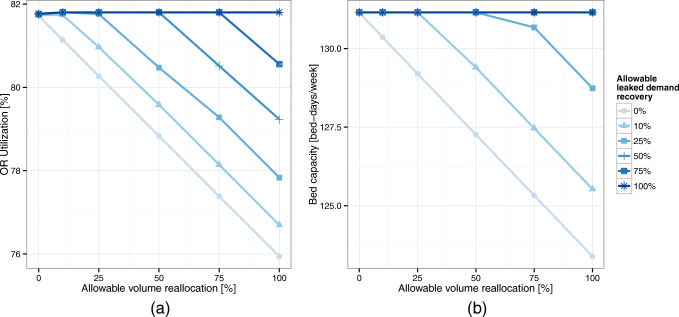
Operating room utilization, and required ward-beds capacity at the AMC when reallocating AMC volumes and recovering unmet demand

In terms of bottleneck resources, the operating room and ward-beds capacity utilization (see Fig. 9) –as well as preoperative and post-anesthesia bays– decrease as more flexibility (reallocation) is permitted. Figure 9a shows the changes in the AMC’s operating room utilization; it decreases with the reallocation of existing volumes but increases again with the recovery of unmet demand. Interestingly, the ward-beds resource seems to be the most limiting resource for the recovery of unmet demand. In Fig. 9b, we observe that the ward-beds need reaches the baseline capacity (26.23 beds per day) when 50% of the unmet demand is recovered.

**Fig. 10:**
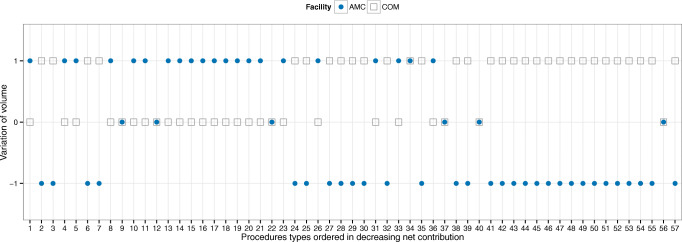
Changes in the network’s portfolio of services obtained by recovering unmet demand. Note: We assume up to 10% volume reallocation from the AMC to the community and full unmet demand recovery across the network. Directional volume changes are measured relative to the baseline volumes. Positive changes are coded as ‘ + 1’, negative as ‘− 1’, and no change as ‘0’. The AMC is represented by filled circles and the community hospitals (combined) as unfilled squares. When there is no change in volume (AMC and COM at zero), it means that there is no unmet demand for the specific service

In terms of volume, this is now redistributed between the community and the AMC. For example, let us assume that reallocation is allowed for up to 10% of AMC volumes and that all leaked demand can be recovered across the network. Figure 10 summarizes the directional changes in volumes. As before, services are ranked on the x-axis from highest to lowest ‘net contribution’. The model suggests reallocating to the community services at the bottom of this ranking. This, again, was in agreement with our partner’s expectations. However, the model also suggests reallocating to the community services at the top of the ranking (procedures types 2 (HPB), 3 (Colorectal), 6 (General Surgery), and 7 (HPB)). This was surprising, and somehow counter-intuitive for the executives and managers. The difference is rooted in the fact that these services consume a large number of bottleneck resources (largest and darkest bubbles in the upper middle area in Fig. 5), and also in the fact that the traditional approaches ignore the opportunity cost of resources. Thus, by shifting cases of these services to the community, the model frees up AMC capacity and backfills it with cases of services that, under the traditional view, are seemingly less profitable. Ultimately, the reallocation results in better value out of the entire network capacity.

**Fig. 11:**
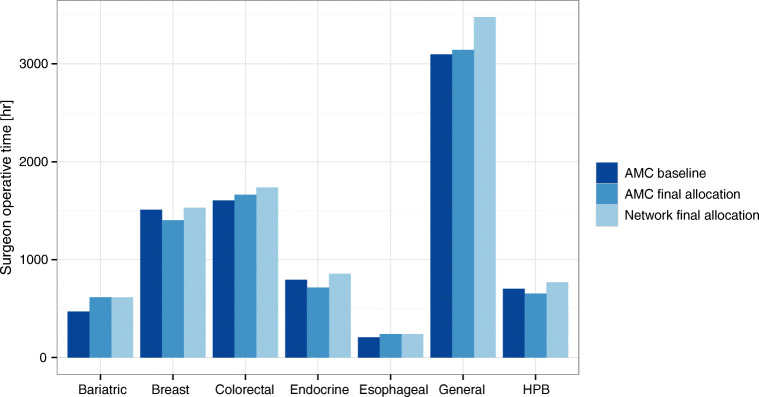
Example of total surgeons’ operating time by specialty at the AMC and across the network. Note: We assume up to 10% reallocation from the AMC to the community and full unmet demand recovery. The ‘AMC baseline’ scenario corresponds to the baseline volumes performed at the AMC. The ‘AMC final allocation’ corresponds to the optimal total operative time at the AMC, and the ‘Network final allocation’ is the total operative time across the entire network (AMC plus COM)

Another output of the model is the allocation of surgeons’ time across the network. In Fig. 11, we show an example of the resulting surgeons’ operative time allocation assuming a 10% volume reallocation from the AMC to the community and full unmet demand recovery. Even in the case where all leaked demand is recovered, the overall surgeon time utilization is below 70% (versus 62% in the baseline scenario). In general, we observe that breast, endocrine, and HPB specialties, and therefore surgeons’ time, are partially moved from the AMC to the community, while the other specialties increase or maintain their presence at the AMC. Interestingly, breast and endocrine specialties have the lowest average profitability per case, and HPB has the highest. Moving HPB cases out to the community setting was, again, somehow counter-intuitive for the managers and executives, but it demonstrated how traditional approaches might fail to capture complex interactions in a network environment. On the other hand, at this point in time, and independently of this study, our partner network started to shift some breast and endocrine services to a recently acquired clinic, our analysis validates such strategy.

### Limitations

The specific results reported in this case study cannot be generalized to other healthcare networks. Recommendations pertaining to case-mix, capacity deployment, and resource allocation at each hospital location reflect the financial and operational realities at our partner system. Reimbursement rates are sensitive to the mix of payers and patients, which can vary widely across institutions. Resources and their usage, as well as demand and growth opportunities, are also specific to each hospital’s reality and practices. Nonetheless, the optimization model and estimation methods can certainly be used elsewhere. Specifically, the methodology used for modeling and estimating resource usage, capacities, demand, revenue, and cost, and the mapping approaches developed to convert from DRGs and ICD-9 to internal codes can be replicated. Because of the ambiguity in how cost allocation and profitability analysis are conducted in practice, others will most likely find surprising insights when comparing the model recommendations to current practice.

Specific to our case study, we consider a static and deterministic view of our partner system operations. Service profitability is an evolving metric that is subject to market changes, such as reimbursement models, contract negotiation, and patient composition (e.g., type of insurance, age, and severity of illness). For instance, it is likely that new financial incentives to move care from inpatient to outpatient or ambulatory settings will result in a different prioritization of procedures [19]. Also, unmet demand recovery, in reality, is a complex phenomenon and would likely depend on other exogenous factors (e.g., competition), as well as the various recovery mechanisms, and their effectiveness, used by the network. Our model does not include retention/growth measures directly. Additionally, the best retention/growth measures might be different depending on the optimal case-mix recommendation (e.g., offering bariatric surgery only at the main campus versus offering it at each satellite location.) Indeed, the output of our model might be used to inform the implementation of such recovery measures.

In this work, we ignore all fixed costs because our focus was on informing short-term tactical decisions, however, to study long-term strategic decisions the model should include fixed costs. But note that using full cost to value services is not recommended. Full cost includes fixed cost allocations that are derived based on current volumes–these cost allocations are likely going to change when the case-mix of services shifts. In addition, using full cost assumes cost will change linearly in volume, which might not be the case as some resources might have economies or diseconomies of scale. Our modeling framework is very flexible and can be easily modified to study strategic long-term resource allocation and case-mix decisions and avoid the shortcomings above. For example, currently, the model assumes a fixed number of nurse-hours available per week, but that capacity might not be appropriate for a long-term strategy. One might want to increase this capacity by hiring additional nurses. The model can incorporate this by considering the nurse-hours as a decision variable and adding its cost to the objective. The dual prices from the short-term implementation of the model can be used to identify the resources that are limiting the output of the process so decision variables should be added for those resources in the long-term implementation. By modeling labor capacity in this fashion instead of through cost allocations to services, we avoid the noise introduced by arbitrary allocation rules, which are often based on uncertain volume projections. Moreover, resource economies/diseconomies of scale can be modeled directly in the cost function. The same approach can be taken for equipment and facility-related resources (e.g., additional beds allocated to a surgical department) whose capacity is flexible. We expect the optimal short-term and long-term strategy to differ, and a priori, it is unclear whether the optimal long-term strategy will resemble the outcome of myopic rules based on full cost allocations–we thus advise to use the model whenever possible.

## Conclusion

In this paper, we propose a general framework to support strategic decision making in health care delivery networks. Specifically, we developed a linear optimization framework akin to revenue management models that allow us to support several strategic network decisions related to case-mix, resource and capacity allocation, and network integration and expansion. Our approach is surprising, in stark contrast with current practices; the contrast lies in how one ‘prices’ the use of resources which must be provisioned well in advance of serving procedures. Existing practice will frequently ‘amortize’ the real dollar cost of these resources across activities in an ad-hoc fashion based on cost accounting principles. Our approach prices each service according to the resources it uses, where resources are valued based on their opportunity cost (i.e., the cost for an additional unit of that resource). This opportunity cost corresponds to the shadow price of the resource and is computed while acknowledging all of the operational constraints one faces in providing services and the demand for those services across the network. This ultimately allows us to provide an objective allocation of resources and capacities in a network environment.

We demonstrate one of the potential applications of our framework by working in collaboration with the surgical department of a network of hospitals. The goal for our partner network was to determine how to better use the spare surgical capacity in the community to recapture leaked demand while maximizing profits across the network. We conducted several analyses that demonstrate how the model outputs differ from traditional practices, specifically, compared to how services are commonly prioritized. The results revealed significant and practical managerial insights to the executives and managers related to case-mix across the entire network and at the hospital level, leaked demand recovery, and allocation of capacity among surgeons and specialties.

More generally, our framework can support other strategic decisions related to how to use a multi-site health care network to meet the population care needs, which procedures to offer at each location, and the corresponding capacity allocations. In terms of resources, our approach can guide decisions on how to allocate limited resources (e.g., operating room time, surgeon time, and specialist time) across the various procedures and activities in alignment with the network’s welfare objectives. Additionally, decisions, such as which growth opportunities to pursue, how much capacity to reserve for specific procedures, and what surgeon’s expertise to bring into the network, can be supported as well. Finally, by incorporating specific operations constraints, our framework can also be used to analyze current operations, identify bottlenecks, and evaluate the effect of changes in capacity, payments, and the portfolio of services offered in the network’s objectives. Overall, we strongly believe that our proposed approach has the potential to transform how health care networks understand the cost and allocate resources in a network environment in practice.

## Appendix A: Notation summary

**Table 3:** Definition of sets and indexes

Set	Notation
Locations	*l* ∈ *L*
Services	*p* ∈ *P*
Activities	*a* ∈ *A*
Fixed resources	*r* ∈ *R*^*F**i**x*^
Flexible resources	*r* ∈ *R*^*F**l**e**x*^
Substitutable resources	*r* ∈ *R*^*S**u**b**s*^, *s* ∈ *S*(*r*)

The subset *S*(*r*) corresponds to a set of substitute resources for resource *r*. The complete set of resources *R* = *R*^*F**i**x*^ ∪ *R*^*F**l**e**x*^

**Table 4:** Model parameters

Parameter	Notation	Description	Units
Revenue net of variable cost	*π*_*p**l*_	(Revenue–service cost) obtained by performing .	Dollars.
		service *p* at location *l*	
Resource usage	*u*_*p**a**r**l*_	Amount of resource *r* required by activity *a* to	Time equivalent units.
		provide service *p* at location *l*.	
Capacity	$c_{rl},\ \tilde {c}_{r}$, and $\hat {c}_{r}$	Amount of fixed, flexible, and substitutable	Time equivalent units.
		resource *r* available at location *l*, or across the entire network.	
Network demand	${{\varDelta }}^{-}_{p}$, ${{\varDelta }}^{+}_{p}$	Minimum and maximum network demand for service *p*.	Cases.
Single hospital demand	$\delta ^{-}_{pl}$, $\delta ^{+}_{pl}$	Minimum and maximum demand for service *p* at location *l*.	Cases.

## Appendix B: Estimation of model parameters

We estimate model parameters based on the current utilization levels at the AMC, and spare surgical capacity in the community hospitals. Specifically, we use historical financial, surgical records, capacity data, and in-network claims data, from the AMC and community hospitals (where available) from years 2009–2013. Most of the parameters are estimated using the last complete year of data (2012). In addition, the IT systems are not integrated across our partner network, hence the AMC’s data bases only contain historical records of surgical patients at this location. Thus, data for the community hospitals had to be collected manually through surveys and interviews. The estimation of the model parameters is specific for this application, but it can serve as a guideline for other applications of our model.

### B.1 Capacity of resources

Limiting the analysis to a subset of services introduces a unique challenge in capacity modeling. Specifically, resources are used by all services, including those that are not part of our model. To reconcile this discrepancy, we approximately segment the capacity into the portion that is available for the studied services. For operating rooms, we have a natural paradigm to assist us in performing this segmentation: surgeon block time. For the AMC, we estimate the *surgeons block time share* in 2012. This corresponds to the portion of operating room total block time assigned to surgeons who can perform the set of studied procedures. We use this share to adjust capacity of various other resources as follows.

#### Capacity for type A resources

The capacity for supplies and equipment is difficult to scale. Cleaning, transportation and set-ups consume usable time. At the AMC, the inventory of resources is readily acquired from the IT system. Thus, we adjust total capacity proportionally to surgeons’ block time share. Even though this is a conservative assessment, it is more realistic than assuming that the entire capacity is available for the studied services. At the community hospitals, on the other hand, such an IT system does not exist, and we had to manually collect data on spare capacity with the assistance of the local nursing teams.

#### Capacity for type B resources

At the AMC, the available operating room capacity is the result of scaling total operating room time by the surgeon’s block time share, and by the *average studied services share*. The latter share corresponds to the average portion of surgeons’ block time that has been historically spent on cases of the studied procedures. The operating room available capacity at the AMC (in time units) is

$$ \begin{array}{@{}rcl@{}} c_{OR, AMC} &=& \text{Total OR block time at AMC in a year} \\ &&\times\text{Surgeons block time share at AMC} (\%) \\ &&\times\text{Avg. studied services share at AMC} (\%). \end{array} $$

We follow a similar procedure for determining available capacity for the pre-operative and post-anesthesia bays. For the recovery beds, on the other hand, we were unable to directly estimate the number of beds available for cases of the studied services, so we instead approximate the available capacity by computing the number of bed hours used by our 57 services in 2012. We use this as the minimum capacity available. For the community hospitals, we directly ask the operating room managers to estimate the spare capacity for all the physical infrastructure resources.

#### Capacity for type C resources

The AMC surgeons operating time available capacity is computed on a surgeon class basis based on the AMC 2012 records. A surgeon class is a set of specialties (bariatric, breast, colorectal, endocrine, esophageal, general, and Hepato-Pancreato-Biliary (HPB)); each surgeon belongs to exactly one class, and two surgeons are in the same class if they can perform surgeries from the same set of specialties. For example, John and Jane specialize in *bariatric* and *colorectal* surgeries. Judy, on the other hand, masters *colorectal* and *endocrine* surgeries. In this example, there are two surgeon classes, {bariatric and colorectal} and {colorectal and endocrine}. John and Jane belong to the first class, and Judy to the second. Thus, each surgeon class is treated as a different resource in the model, and its capacity corresponds to the pooled capacity of the surgeons in the class. Specifically, we first compute the actual total operating time (TOT) spent on the studied services. TOT includes all the operating time, regardless of whether the case was performed within surgeons’ own block time. Secondly, we estimate the adjusted block time (ABT). This is the surgeon’s block time allocation, but scaled by the average share of surgeon’s total operating time spent on the studied procedures, plus any shared block time allocation. Shared blocks are blocks used by a group of surgeons, and we estimate the distribution of this time across surgeons based on historical usage. Again, ABT is a conservative estimate of the time surgeons could spent operating cases of the studied procedures. Finally, surgeon’s available time is defined as the maximum between TOT and ABT. Thus, the available time for surgeon class *r* is just the pooled capacity across the surgeons in the class,

$$ \hat c_{r} = \underset{i \in Surgeon\ Class\ r}{\sum} \max \{TOT_{i}, ABT_{i}\} $$

### B.2 Resource usage

Resources are used by different activities and services, and the usage is measured in time equivalent units and depends on the duration of the activity and the quantity of resource used during the activity, both of which are specific to a service.

#### Typical activity duration

Using historical data from AMC in 2012, we compute the empirical distribution of the duration of each phase in the surgical path (Fig. 2) for each surgical service. For phases I–IV, the duration differs in variability, and right skewness. Thus, we use the median duration as the typical phase duration. For phase IV however, we noted that some services are performed in both, inpatients and outpatients settings. Using median duration for phase IV does not represent the typical duration for neither subset of patients. Instead, we use the weighted average of the median duration for the two subset of patients to estimate the duration of phase IV.

$$ \begin{array}{@{}rcl@{}} \text{Typical duration}_{\text{Phase IV}} \!&=& \frac{\text{Inpatient cases}}{\text{Total cases}} \\&&\times \text{Inpatient median length-of-stay.} \end{array} $$

Finally, we also assume that the duration of phases I–IV is the same across all locations. Conversely, the duration of the turnover (phase V) is not service specific but location dependent, and it is defined according to the scheduling standards at each hospital (e.g., 30 min at the AMC, 45 min at the community hospitals).

#### Typical resource quantity

Using resource usage data from AMC in 2012, the typical resource quantity is modeled by the average quantity used in each phase of the surgical path. Although our general model allows for different resource usage at each location, we assume that the typical quantity is the same across hospitals. According to the local nursing teams, required resources and quantities are inherent to the service, and are not expected to change significantly based on location. Differences in resource requirements are exceptions, and we handle them individually. Thus, the typical resource usage (*u*_*p**a**r**l*_ in the general formulation) is defined as follow, for a given phase *a*, the typical usage of resource *r* by service *p* is

$$ \begin{array}{@{}rcl@{}} u_{par} =\text{Avg. resource \textit{r} used by service \textit{p} in phase \textit{a}}\\ \times \ \text{Typical duration of phase \textit{a}} \end{array} $$

The index *l* is dropped since in the application we assume that usage is the same across locations.

### B.3 Demand

Unmet demand, or leaked demand, is estimated by analyzing claims data of in-network patients that received surgical care outside the network in 2012. The leaked demand records are indexed by the Diagnosis-Related Groups (DRGs) coding systems; the HCFA/CMS-DRG for Medicare cases and the AP-DRG for non-Medicare cases. The mapping between this coding system and the hospital internal coding system is non-trivial because multiple DRGs can be assigned to a single service type. To reconcile this, we create a mapping based on the empirical frequency of DRGs on existing demand. Consider a service *x* and an DRG code *z*, then using the AMC existing volumes, we can estimate the probability of having a service *x* given that DRG *z* is observed as

$$ P(x|z)=\frac{P(x,z)}{P(z)} \sim \frac{\text{No. cases of \textit{x} and \textit{z}}}{\text{No. cases of \textit{z}}} $$

Thus, the unmet demand for a specific procedure type *x* is simply the weighted average of the leaked demand volumes

$$ \text{Unmet demand(\textit{x})}=\underset{z \in DRG}{\sum} P(x|z) \times \text{Leaked demand(\textit{z})} $$

### B.4 Revenue and cost

A major difficulty in the estimation of revenue and cost per service is that financial data is commonly indexed by encounter (i.e., entire episode of care), and itemized using a different coding system, usually ICD-9 or ICD-10. A single episode of care may involve different surgical services and medical services (which are not included in this application). We are able to parcel out the codes related to surgical services, but there is still the problem that multiple codes, and not necessarily the same codes in all cases, are linked to each surgical service. To address this, we create a mapping based on the empirical frequency of ICD-9 codes and services. For each encounter, we can identify a major surgical service, and a primary ICD-9 code. Based on these, we consider a service type *x*, and a primary ICD-9 code *y*, and estimate the conditional probability of observing service *x* given that the primary ICD-9 code *y* was recorded as

B1 $$ P(x|y)=\frac{P(x,y)}{P(y)} \sim \frac{\text{No. encounters of \textit{x} and \textit{y}}}{\text{No. encounters of \textit{y}}} $$

We were able to estimate these probabilities using the AMC financial data from 2012. Thus, the average revenue of a specific surgical service is simply the weighted average of the average payments by primary ICD-9 codes:

B2 $$ \begin{array}{@{}rcl@{}} \text{Avg. Revenue AMC(\textit{x})}= \\ \underset{y \in \text{ICD-9}}{\sum} P(x|y) \times \text{Avg. AMC Payment(\textit{y})} \end{array} $$

For the community, however, estimating the probabilities in Eq. B1 is not possible. The payment data is also indexed by encounter and ICD-9 code, but it does not indicate the major surgical service. Instead, we use the AMC probabilities as a proxy. However, even if we use the mapping from the AMC, we still have the problem that some ICD-9 codes are rarely performed in the community, or not at all, thus the average payment per ICD-9 in the community is noisy. To overcome this, we adjust the average payment of ICD-9 codes that have less than 10 observations using the AMC’s average payment. Thus, for an ICD-9 code *y* with sample size *n* < 10

$$ \begin{array}{@{}rcl@{}} \text{Adj. Avg. COM payment(\textit{y})} = \\ \text{Avg. COM payment(\textit{y})}\ \left( \frac{n}{10}\right) + \\ \gamma \text{ Avg. AMC payment(\textit{y})}\ \left( \frac{10 -n}{10}\right) \end{array} $$

Where *γ* > 0 is the average payment differential between the community, and the AMC, across ICD-9 codes with 10 or more records. For example, *γ* = 0.8 means that the payments in the community are on average 20% lower than at the AMC. We use the adjusted average back in Eq. B2 to obtain an estimate of the average revenue per service in the community.
